# Agency and Anxiety: Delusions of Control and Loss of Control in Schizophrenia and Agoraphobia

**DOI:** 10.3389/fnhum.2016.00459

**Published:** 2016-09-26

**Authors:** Shaun Gallagher, Dylan Trigg

**Affiliations:** ^1^Department of Philosophy, University of MemphisMemphis, TN, USA; ^2^Department of Philosophy, Faculty of Law, Humanities and the Arts, University of WollongongWollongong, NSW, Australia; ^3^Department of Philosophy, University CollegeDublin, Ireland

**Keywords:** sense of agency, sense of ownership, attribution of ownership, schizophrenia, agoraphobia

## Abstract

We review the distinction between sense of agency and sense of ownership, and then explore these concepts, and their reflective attributions, in schizophrenic symptoms and agoraphobia. We show how the underlying dynamics of these experiences are different across these disorders. We argue that these concepts are complex and cannot be reduced to neural mechanisms, but involve embodied and situated processes that include the physical and social environments. We conclude by arguing that the subjective and intersubjective dimensions of agency and ownership cannot be considered in isolation from one another, but instead form an interdependent pairing.

## Introduction

The sense of agency (SA) may be considered an experiential aspect of the embodied nature of self. One way to grasp the role played by SA is to situate it within the context of anomalous bodily experiences. Research on this theme has focused especially on cases of schizophrenia and depersonalization (e.g., Gallagher, 2005; David et al., 2008; Jeannerod, 2009; Sierra, 2009). In cases of schizophrenic delusions of control, according to some accounts, the sense of self-agency is disrupted; the patient at times experiences her thoughts, actions, and bodily movements as controlled by another agent. Importantly, the disruption of SA occurs not simply in an intra-psychic manner, even in the case of thought insertion, but involves relations to others and the world more broadly (Gallagher, 2012).

Alongside schizophrenia, agoraphobic anxiety offers another way in which disruptions in the experience of agency reveal the dynamic and relational structure of this phenomenon^1^. There are at least two points to consider. First, in terms of first-person experience, subjects with phobic anxiety tend to mistrust their own response to the world, feeling their bodies could give way at any point, thus positioning the locus of control outside of selfhood. Second, in cases of agoraphobic anxiety, loss of SA leads to a partial loss of the sense of bodily ownership. This can also be understood in the context of intersubjective relations. For the agoraphobic person, the encroachment of other people into one's own space leads to anxiety, generating an experience of the body as both my own and not my own concurrently (Trigg, 2013a).

In this paper, after reviewing the distinction between SA and sense of ownership (SO), we explore these notions, and their reflective attributions, in schizophrenic symptoms and in agoraphobia. We show how the underlying dynamics of these experiences are different across these conditions. We conclude by arguing that the subjective and intersubjective dimensions of agency and ownership cannot be considered in isolation from one another, but instead form an interdependent pairing.

## Sense of agency and sense of ownership

A number of theorists have defended clear phenomenological distinctions between experiences of agency and ownership (Graham and Stephens, 1994; Gallagher, 2000a,b, 2012; Stephens and Graham, 2000; Tsakiris et al., 2007; Synofzik et al., 2008). These distinctions have been made in regard to both pre-reflective and reflective consciousness. On the pre-reflective level of experience, SA is the sense that I originate and control my actions; SO is the sense that I am the one who is moving or undergoing an experience (Gallagher, 2000a). The case of involuntary action makes the distinction clear. For example, if someone pushes me from behind, my experience is that I am the one moving (I have SO for my bodily movement), but, at least in the first instant, I do not have SA for the movement since I was not the one who initiated the action. The phenomenological claim is that SA and SO are common features intrinsic to most pre-reflective agentive experience. This applies to thinking as well, insofar as thinking is considered to be an action.

Experimental studies have attempted to identify the neural correlates of SA and SO, which are thought to involve correlations between efferent signals (for SA) and afferent signals (for SO) (e.g., Tsakiris and Haggard, 2005), and may involve sensory integration in the anterior insula (e.g., Farrer and Frith, 2002). In a more recent study Tsakiris et al. (2010) found independent activations in midline cortical structures associated with SO, absent for SA; and activation in the pre-SMA linked to SA, but absent for SO. Although this finding supports an “independence” model, where SA and SO are understood to be two “qualitatively different experiences, triggered by different inputs, and recruiting distinct brain networks” (Ibid, 2740), there is behavioral and phenomenological evidence for a more integrative or “additive” model where SA and SO are strongly related (e.g., Caspar et al., 2015).

In addition to pre-reflective SA and SO, Stephens and Graham (2000) have proposed that one can attribute agency and ownership retrospectively based on a judgment of consistency between one's actions (or thoughts or beliefs) and one's self-narrative. They distinguish between the reflective attribution of agency (AA) and the reflective attribution of subjectivity or ownership (AO). They argue that with respect to agency, if an action I perform or a thought that I have are inconsistent with how I understand myself, my introspective sense that I am the agent may be less than if that action or thought is consistent with my self-understanding.

As Synofzik et al. (2008) indicate, AA and AO involve *judgments* of agency and ownership, the result of a second-order reflective consciousness, as distinct from a first-order pre-reflective *experience* of SA and SO. Graham and Stephens suggest that AA involves a process of comparing action (or belief) and narrative to test for consistency. It seems possible, however, that a second-order retrospective judgment about agency may be based directly on the first-order experience of SA (Bayne and Pacherie, 2007; Haggard and Tsakiris, 2009). That is, if I am asked whether I have engaged in a particular action, my reflective stance may simply discover that my pre-reflective experience of that action already involved SA. In that case AA may simply be a report on SA rather than a comparative judgment about one's action and one's self-narrative.

The phenomenological claim that SA and SO are common features intrinsic to most pre-reflective agentive experience has not gone unchallenged, however. Bermúdez (2011), for example, despite his contention that first-person bodily experience counts as a form of self-consciousness, argues that there is no evidence that SO is a feature of pre-reflective experience; he considers the SO to be a product of reflective judgment, which would make it equivalent to AO. Bermúdez interprets claims about SO to be claims about an aspect of experience separate and distinct from proprioception, kinaesthesia and other bodily sensations, and he denies that there is any such aspect. In contrast, we understand SO to be an implicit aspect of proprioception and other bodily sensations, rather than something separate from them (Gallagher, 2005; also see de Vignemont, 2007, 2013). In fact, this implicit self-experience (or ipseity) is precisely what makes first-person bodily (proprioceptive, kinaesthetic) awareness itself (i.e., prior to any judgment) a form of self-consciousness—it's what puts the “proprio” in proprioception. On this view such experiences are characterized by a “perspectival” SO (Albahari, 2006), i.e., an intrinsic SO directly tied to a first-person perspective.

With respect to SA, Grünbaum (2015) offers a more detailed critique that draws a conclusion similar and parallel to Bermúdez's conclusion about SO, namely that there is no separate and distinct pre-reflective SA that acts as the basis for a judgment about agency (AA). Grünbaum focuses on the particular account of SA that considers it the product of comparator mechanisms involved in motor control. He doesn't deny that a comparator mechanism may be involved in motor control, but he challenges the idea that comparator processes generate a distinct experience of agency. He views the claim that SA is generated by such mechanisms to mean that SA is intention-free. By “intention-free” we take him to mean that, on such accounts, SA is generated even if the agent has not formulated a prior, personal-level intention to act in a certain way. Reaching for my cup of tea as I work on my computer does not require that I consciously deliberate and form a plan to do so. Still, it counts as an intentional action and may involve a present intention-in-action, and motor intentions (Pacherie, 2006, 2008). Moreover, at least some comparator models include the idea that there is some functional element in the system that counts as an intention, and that this intention is compared to efference copy or sensory input from the movement to facilitate motor control (e.g., Frith, 1992; Wolpert and Flanagan, 2001). In this respect it's not clear that SA can be characterized as intention-free.

Grünbaum also points to an important qualification involved in a number of experiments on SA. For example, in an action-recognition experiment, Daprati et al. (1997) ask subjects to perform a hand movement and monitor it on a computer screen, which shows either their own hand movement, or a hand movement made by someone else. They are then asked whether what they saw was their action or not. Typically subjects mistook the actions of the other's hand as their own in about 30% of the cases; schizophrenic subjects who had a history of delusions of control and/or hallucinations misjudged 50–77% of the cases. The problem, as Grünbaum notes, is that on each trial the subject is in fact engaging in the action of moving his own hand. The same objection can be raised in regard to other experiments. For example, Farrer and Frith (2002) use a similar experimental design and claim that this allows for the dissociation between SO and SA. SO for the movement, they contend, was kept constant because the subject moved on each trial; but SA varied depending on whether subjects felt they were in control of what was happening on the computer screen.

With respect to the Daprati et al. experiment, Grünbaum concludes that rather than reporting SA based on comparator processes (since hypothetically that would also remain constant across all trials), subjects were simply reporting differences in what they were monitoring on the screen. An alternative conclusion, however, is that the pre-reflective SA is more complex than an experience that is generated by comparator processes. The idea that SA involves at least two aspects—one having to do with the control of bodily movement in action, and one having to do with the intentional aspect of the action, i.e., what the action accomplishes in the world—has been either assumed (as in Farrer and Frith, 2002) or explicitly stated (Gallagher, 2005, 2012; Haggard, 2005). Even if Grünbaum were right about comparator mechanisms not generating SA, SA may still be generated in our perceptual monitoring of what our actions are accomplishing in the world. Langland-Hassan (2008) raises similar worries about the positive phenomenology of SA, but concludes that the phenomenology of agency is “one that is embedded in all first order sensory and proprioceptive phenomenology as diachronic, action-sensitive patterns of information; it does not stand apart from them as an inscrutable emotion” (p. 392).

Although we cannot respond to all of Grünbaum's detailed arguments here, we do want to indicate that we take SA to be a more complex phenomenon than just a simple phenomenal experience generated by a subpersonal comparator mechanism. Indeed, there are reasons to question whether comparator models of motor control offer the best explanation (see, e.g., Synofzik et al., 2008; Friston, 2011). That issue aside, however, the pre-reflective SA may be constituted by a number of contributories, including reflective processes that involve prior or distal intentions, long-term intentions, and retrospective attribution (Pacherie, 2006, 2008; Gallagher, 2012; Vinding et al., 2013).

It may sound strange to suggest that reflective processes that involve prior intention formation may contribute to a pre-reflective experience of agency. The idea is simply that if I deliberate and create an action plan or prior intention to do something (for example, to buy a new car next week), when the time comes and I put that intention into action, the fact that I had planned it out and am not acting in a completely spontaneous way should enhance my sense of control over my action. If, in contrast, I found myself in the car dealership due to a spontaneous desire for a red Mustang convertible that I spotted on the lot, I might in fact feel a little out of control, and this feeling of lack of control (or decreased SA) may be reinforced when I start to evaluate my action in terms of my self-narrative or in terms of a violation of my long-term intention to reduce my dependency on fossil fuels. In this respect, a lack of deliberative reflection or a modulation in AA, the judgment or AA that I make about my action, may in fact have an effect on my ongoing pre-reflective SA for the action^2^.

On this view we can identify several different contributories to a complex SA connected with any particular action, and we can think of these contributories as forming a dynamical, relational gestalt of factors, changes in any one of which can modulate the experience of SA.

- Formation of prior intentions, often involving a prospective reflective deliberation or planning that precedes some actions.
- Pre-reflective perceptual monitoring of the effect of my action on the world in terms of specific intentional or means-ends relations in specific situations.
- Basic efferent motor-control processes that generate a first-order experience linked to bodily movement in and toward an environment.
- The retrospective AA that follows action.

We want to go even further in identifying contributories to SA, although we won't be able to lay out the entire argument here. We contend that the experience of agency is not reducible to neural comparator mechanisms, even if these mechanisms are involved in motor control, or even to the purely internal processes described above. Rather, we suggest that agency, and the SA that accompanies it, are fully embodied and situated (Buhrmann and Di Paolo, 2015). That it is embodied should be obvious since it involves bodily action, the peripheral nervous system (proprioception, kinaesthesia), autonomic and vestibular processes, affective and emotional aspects^3^ and so on (and on embodied cognition views, even thinking involves bodily processes). That it is situated means that our agency, and our experience of it, can be modulated—increased or decreased—by physical and spatial features of environments as well as social environments that include, not only other people, but also normative, social, and institutional practices (Gallagher, 2012, 2014). Consider that even large social structures (e.g., institutions of apartheid and slavery) can literally rob individuals of their agency and make them feel that they have no control over their own lives (Gallagher, 2011, 2012). Just as spontaneous decisions (e.g., the lack of reflective deliberation, as in the case of spontaneously deciding to buy a car) can diminish one's feeling of self-control and SA as one engages in a particular action, so also a social structure (or intersubjective relation) that takes away the possibility of making one's own decision can have an effect on one's agentive experience in so far as actions may be prevented (by psychologically undermining motivation) or forced (by physical discipline)^4^.

If SA may be modulated by changes in varied factors that involve, most centrally, pre-reflective perceptual monitoring of the effects of action, and basic motor-control processes, but also reflective deliberation, retrospective judgments, environmental, intersubjective, socio-normative, and even cultural and political arrangements, then a disruption in any of these factors may generate nuanced and in some cases, pathological differences in SA of different sorts [as well as in SO, and the attributions of agency (AA) and ownership (AO) in so far as these are related to SA]. That is, we should expect that in different pathologies, SA may be changed or undermined in different ways, depending on what factors may be involved. Accordingly, we turn now to examine changes in SA in two different disorders, schizophrenia and agoraphobia, to discover both what is common and what is different in these disorders with respect to SA.

## Delusions of control in schizophrenia

In typical cases of involuntary movement, efferent signals are missing and, in some situations, so is SA; but SO for the movement is maintained because of the presence of afferent sensory feedback. In such cases, my experience is that I am moving, but I did not initiate the movement. The same logic may explain some aspects of schizophrenic delusions of control. If there are neurological problems with efference copy (understood as a signal sent to a forward comparator involved in motor control) it may result in a loss of SA for the action (Frith, 1992). Consider the following report by a patient suffering from delusions of control.

They inserted a computer in my brain. It makes me turn to the left or right. It's just as if I were being steered around, by whom or what I don't know. (Cited in Frith et al., 2000, p. 358).

The patient expresses no question about who is being turned or steered (he has an intact SO—it is he who is moving); but his experience is of something (or someone) else controlling his movement (he has no SA or sense of self-agency for that movement). This suggests a bottom-up, empiricist account of one aspect involved in delusions of control; something goes wrong at a neural level that has an anomalous effect at the level of awareness.

With respect to the schizophrenic symptom of thought insertion, however, it's not clear that (or how) efference or a comparator model could be involved in thinking, and accordingly, it's not clear that thought insertion can be explained in the same way as delusions that involve bodily movement (Gallagher, 2004). There may be a more general or basic disruption of neuronal processes that affect not just SA for motor action, but also for cognitive processes, resulting in symptoms of thought insertion. SA for higher-order cognitive processes may depend on the anticipatory aspect of working memory (Gallagher, 2000b, 2004), something that may also malfunction in schizophrenic subjects with delusions of control (see Singh et al., 1992; Daprati et al., 1997; Vogeley et al., 1999). Moreover, as we indicated in the previous section, multiple factors may be involved in generating and maintaining SA, and some of these may still remain in effect.

In addition, the absence of efference copy does not explain the full phenomenon of delusions of control since the anomalous experience may also feel alien and there is usually an AA to another person or object. Billon and Kriegel (2014) suggest that rather than there being “something missing” (i.e., SA), delusions of control and thought insertion really involve “something added”—namely a phenomenology of alienation, which is reflected in the subject's claim that someone or something else is making his thoughts. One possible explanation for the alien feeling is that a disruption in the integration of somatosensory signals, visual and auditory signals, and efference (corollary discharge), or some other kind of malfunction in the anterior insula or the right inferior parietal cortex (Farrer and Frith, 2002), or in mechanisms that allows for the proper discrimination between self and non-self (Georgieff and Jeannerod, 1998), may generate a sense of alien control at the level of first-order experience (de Vignemont and Fourneret, 2004; Gallagher, 2004; Pacherie et al., 2006).

On phenomenological approaches to schizophrenia, delusions of control are considered disorders of basic self-experiences. Parnas and Sass (2011) refer to this as a form of *ipseity-disturbance*. Ipseity “refers to the most basic sense of selfhood or self-presence: A crucial sense of self-sameness, fundamental (thus nearly indescribable) sense of existing as a vital and self-identical *subject* of experience or *agent* of action” (Sass, 2014, p. 6). Sass, for example, argues that in cases of ipseity disturbance in schizophrenia, first-person experience is disrupted in two central ways. First, the patient engages in “hyper-reflexivity,” which is marked by an amplified self-consciousness of processes and phenomena that would normally be tacit, or “inhabited” as part of oneself (Sass, 2014, p. 6). Such self-consciousness, Sass notes, is neither introspective nor reflective in nature, but instead functions in a perception-like way. Second, a “diminished self-affection” emerges, such that the patient undergoes a diminished “sense of existing as a subject of awareness or agent of action” (p. 6) and a feeling of alienation. According to this model, an initial heightened self-awareness leads to a diminished self-awareness and alien feeling, similar to the way in which if you stare at the back of your hand long enough it starts to feel as if you are staring at something that is not you^5^. The ipseity-disturbance model is grounded in the broader distinction between a minimal or prereflective sense of self-experience and a second-order, reflective level involving the narrative of autobiographical self (Sass, 2014, p. 7; Gallagher, 2011). The disturbance at stake in schizophrenia is a disturbance leveled precisely at the prereflective or minimal self, rather than the narrative self (Parnas and Sass, 2011). What is at stake, therefore, are the experiential aspects of SA and SO.

In contrast to empiricist and phenomenological explanations, Graham and Stephens (1994) and Stephens and Graham (2000) propose that in cases of schizophrenic delusions of control or thought insertion the problem is with AA. The subject fails to attribute agency to his actions or thoughts because they seem radically inconsistent with his self-narrative. In such cases the important change is in the reflective judgment of agency. According to Graham and Stephens, there is no change in AO, however; the subject does not deny that the action is being carried out by his own bodily movement, or that the thought is occurring as part of his own experience. Indeed, that is precisely his complaint—that this action or thought involves his body or his thinking, but does not seem consistent with his beliefs or self-conceptions. Again, although this top-down, rationalist account may be more consistent with the view that a delusion is “a false belief based on incorrect inference” (as it had been controversially defined by the DSM-4), it doesn't provide a full explanation since it's not clear why an inconsistency between action and narrative would prevent an attribution of self-agency rather than, for example, a sense that one has made a mistake or was simply inconsistent in one's actions, or why it would motivate a misattribution of agency to someone else. In addition, top-down accounts don't address a puzzle raised by Bayne and Pacherie (2004,p. 8): “We are also puzzled by the question of how a top-down account of delusions could explain the damage to the autonomic system that one finds in the Capgras and Cotard delusions. Is this caused by the delusional belief? That seems unlikely.” More generally, top-down accounts don't provide a clear picture of how organic malfunction is related to the cognitive mechanisms that purportedly generate delusions.

Furthermore, if introspective or narrative capabilities are in some way undermined by organic damage, as Graham and Stephens would have it (also Campbell, 2002), it is not clear why the subject's delusions would be selectively about certain topics and not others—that is, why the subject is not delusional about everything he believes, or why some actions or thoughts are considered alien, but not others. This has been called the problem of specificity (Gallagher, 2004, 2007). Pacherie et al. (2006, p. 575) suggest that it remains unsolved. This also seems a problem for those empiricist (bottom-up) accounts that would explain the loss of SA solely in terms of a faulty comparator. It may be that the specificity of delusions depends on a kind of internal logic that involves the integration or disintegration of selfhood; and a solution may also depend on conceiving of SA as constituted by a plurality of factors that include physical and social environmental elements.

A more hybrid explanation may also address some of these issues. Two-factor models of delusion combine top-down and bottom-up accounts and suggest a more central role for neurological problems. The first factor consists of an anomalous experience, such as an odd feeling (or lack of appropriate feeling), anomalous perception, or hallucination caused by some neurological dysfunction that interferes with SA or with some emotional aspect of experience; the second factor consists of an attempt to explain or rationalize the anomalous experience, leading to what the DSM-5 defines as “fixed beliefs that are not amenable to change in light of conflicting evidence” (see, e.g., Ellis and Young, 1990; Davies et al., 2001; Garety et al., 2001). On some views, experience itself is considered delusional, while higher-order cognition simply reports (endorses and, as things develop, perhaps enhances) the delusion (see e.g., Hohwy and Rosenberg, 2005; Mundale and Gallagher, 2009).

Not everyone agrees, however, that the experience or judgment about agency is the thing at stake in delusions of control and thought insertion. Bortolotti and Broome (2009), for example, deny that delusions of control and thought insertion involve problems of SA or AA. Rather, they propose that such delusions involve problems with AO, attributions of ownership. They view this as a “more demanding notion of ownership” that involves a self-ascription condition by which a subject acknowledges an action or thought as her own and ascribes it to herself on the basis of introspection, or her reasons (or lack of reasons) for acting or thinking in that way. That there may be a problem with AO in such cases, however, does not rule out the possibility that the primary problem is still a problem with SA. If we ask why the subject reflectively disowns the action or the thought, two answers still seem possible. Either (1) the thought doesn't fit with her self-narrative (as suggested by Graham and Stephens), and is not “endorsed” by the subject since she is not able to provide reasons for it (as suggested by Bortolotti and Broome), or, (2) the action or thought actually feels or is experienced as alien—a first-order experience that may have initially motivated the second-order reflection. This type of first-order experience, even in the case of thought insertion may involve bodily and spatial aspects, as when patients describe thoughts entering into their heads literally at certain locations on their skulls (e.g., Cahill and Frith, 1996). Moreover, this first-order feeling of alienation may result from, or may result in, a modulation or displacement of SA for that action or thought. So, even if Bortolotti and Broome are right that the person's second-order, retrospective report indicates a problem with AO, this problem may be due to a first-order, experiential problem with SA and/or feeling of alienation (Gallagher, 2015).

Billon (2013), who also thinks that thought insertion involves problems with AO, provides a different argument against the SA explanation. He argues that the subject doesn't actually have a first-order experience of the thought. Rather, the inserted thought is generated and inserted by the second-order reflection. Such thoughts lack first-order (pre-reflective) phenomenality and therefore there can't be a first-order SA or SO for the thought.

Even if one were to accept this account, however, the problem is still one that involves SA. What does it mean to be able to come upon a thought that is in itself unconscious (without phenomenal properties) and for that reason, seemingly not mine? Billon's analogy is that the inserted thought is “akin” to a sentence uttered by someone else, or in a text that one is reading. I may have reflective access to the thought in a way that is akin to my perceptual awareness of a sentence on the page or of the sentence you just uttered. There may be something it is like to have that reflective access, or to perceive the sentence, but the sentence, does not have anything like a thought-like phenomenal character. I come upon a certain intentional content, a certain thought-meaning that seems independent of any process of thinking that would bestow on it a phenomenal feeling such that it would feel like I was the one thinking it. In that case it's a thought that I seemingly did not think—something that did not get generated in my thinking process. But if what's missing is the sense that I am the one generating the thought in a process of thinking, then what's missing is precisely the sense of self-agency—the pre-reflective SA which just is the sense that I am the generator of the thought or action.

The analyses of Bortolotti and Broome and Billon suggest again the complexity and ambiguity involved in these issues—that is, the complexity and ambiguity involved in the actual relations that exist among SA, SO, AA, and AO. It's possible that SA and SO are closely related on the prereflective experiential level of ipseity (consistent with an *ipseity-disturbance* model—Parnas and Sass, 2011; Sass, 2014) even if they are not correlated to the same neural activations (Tsakiris et al., 2010); and it's also possible that there are reciprocal relations between SA/SO and reflective judgments (AA and AO) about agency and ownership so that modulations run in both directions. If, for example, SA is disrupted by neural or extra-neural factors, both AO and AA may be affected such that I am led to judge an action or thought as not mine or as not under my control. This ambiguity is reflected in the various explanations of schizophrenic delusions of control and thought insertion.

## Loss of control in agoraphobic anxiety

The experience of agoraphobic anxiety presents us with an interesting counterpart to that of schizophrenia. If schizophrenia tends to involve a disruption in SA, which may also involve a disruption in AO, such that one experiences “a disturbance in the ownership of one's body, thoughts and actions, accompanied by faulty self-monitoring” (Park and Nasrallah, 2014, p. 1), then in the case of anxiety, the disruption in agency may play a different role. Unlike schizophrenia, which can entail a “severe erosion of minimal self-experience or real confusion of self and other” (Sass, 2014, p. 5), in cases of agoraphobic anxiety, the boundary between self and other tends to be retained. Indeed, it is precisely because this boundary is retained rather than destroyed that anxiety and a loss of control emerges. In this section, we attend to this loss of control in and through the experience of the anxiety as it relates to agoraphobia. Our intention in this section is to further underscore our view that dimensions of agency and ownership are intertwined, and always situated within both a subjective and intersubjective context. Our secondary aim is to consider the points of convergence and divergence between agoraphobic anxiety and schizophrenia.

Agoraphobia presents us with an especially clear (and often striking) sense of how a disruption in agency can lead to a disruption in a sense of self more broadly. This is clear in at least two ways. In the first case, the anxiety specific to agoraphobia often involves a disturbance in bodily motricity, such that sensations of anxiety, including the inability to move or the sudden urge to move, is felt as if it comes from nowhere. In the second case, the body of the agoraphobic person is often presented as a distinct *thing* in the world rather than a center of agentive selfhood or a body-as-subject, thus disturbing SO, or more precisely, the felt sense of bodily ownership. Together, we consolidate these aspects under the heading of the *bodily-inhibition model of anxiety*. Such a model allows us to see that what is at stake in agoraphobic anxiety is not simply the discomfort of physical sensations or symptoms, but instead the threat these symptoms pose to the integrity of self and self-agency. To defend this claim, we begin by detailing the agoraphobic condition before considering its conceptual implications for an understanding of agency.

In clinical terms, agoraphobia tends to be characterized by symptoms such as heart palpations, trembling of the legs, nausea, social discomfort, fear of losing control, a sense of impending doom, and an alienation from the body. Etymologically, agoraphobia is situated in relation to public spaces the word stems from *agora* (Greek for marketplace) and *phobia* (from the Greek word *phobos* meaning flight or terror) (Goldstein and Chambless, 1982). According to Carl Westphal, the originator of the term “agoraphobia,” the anxiety experienced during an attack of agoraphobia was often alleviated when the agoraphobic person was accompanied by a trusted companion, was slightly intoxicated, or was able to use a “prop” to move around the world, such as a stick or an umbrella (Knapp, 1988).

In causal terms, Westphal accented a fault in thinking, remarking that, the problem is “more in the head than in the area of the heart” (p. 60). From the perspective of a cognitive model, the idea is that our thinking is at fault, specifically thinking orientated toward dangers in the surrounding world. According to this model, three stages can be mapped out, each of which delineates the development of agoraphobia (Clark, 1988). In the first stage, a subject perceives a threat in the environment, which seems more dangerous than it actually is in objective terms. This environmental danger can also be reflected in bodily sensations. Thus, where panic based agoraphobia is concerned, the tendency to perceive threats in the environment is transferred to a “misinterpretation” of specific bodily sensations, in which those sensations are regarded as signals of impending disaster (Clark, 1988). Such sensations include the sense of impending collapse, a loss of control, an anxiety over passing out, and a more generalized anxiety over “losing one's mind” (Barlow, 2002, p. 107). In the second stage, once these sensations become marked as a focal point in a subject's experience, an adjoining coping and behavioral mechanism forms, which is orientated toward the avoidance of places that arouse undesirable sensations. Thereafter, a vigilant mode of anticipating both the onset of anxiety and possible “threats” becomes a defining feature of the agoraphobic person's world. In the final stage, to counter these threats, avoidance behavior becomes habitual, a way of organizing both the social and spatial dimensions of a subject's world, such that the chance of experiencing panic or anxiety is minimized. Of course, this “mastery” over anxiety comes at the expense of the subject's freedom and an experienced loss of SA. In this respect, Isaac Marks provides a succinct account of the development of agoraphobia: “Once she cannot get off an express train, as soon as anxiety starts she will restrict herself to local trains; when these, too, become the setting for anxiety she retreats to buses, then to walking, then to going only a few yards from home, until finally she becomes unable to proceed beyond the front gate without a companion” (Marks, 1987, p. 336). What starts out as taking action to restrict one's actions in certain places and contexts, ends up in a feeling that one cannot take action at all in those places and contexts.

*Prima facie* this cognitive model of the development of agoraphobia suggests that it can be efficiently treated by cognitive oriented behavioral therapy (CBT) (Meyer and Gelder, 1963, p. 19). One reason for the relevance of CBT is that symptoms of agoraphobia present themselves as discrete events in what is often otherwise a functional existence. Using CBT, patients are educated about the physiological processes that give rise to an acute sense of anxiety. Once the subject “accepts” that their anxiety is a misinterpretation of perceived danger, “the secretion of adrenaline” is diminished thanks to a “cognitive restructuring” (Aslam, 2012)^6^. This suggests that, in contrast to schizophrenia, the subject may be able to reflectively alter his belief structure and adjust his behavior. The person with schizophrenic delusions, according to the DSM, holds fixed beliefs that are not amenable to change in light of reflectively considering evidence to the contrary. CBT treatment of agoraphobia is often implemented alongside exposure therapy, where the patient is encouraged to desensitize themselves to places and situations that are liable to invoke and provoke anxiety (Edelman and Chambless, 1993). Patients are then asked to repeat the procedure in order to facilitate and expedite the desensitization process, until the patient is entirely acclimatized to the fact that the places originally thought of as terrifying are, in reality, devoid of danger. As a result, the patient is able to inhabit the world without the sense of impending collapse previously associated with venturing outside the home.

Recent studies have focused on identifying the neural correlates of agoraphobia with the intention of predicting treatment response to CBT (e.g., Lueken et al., 2013; Hahn et al., 2015). In contrast to clinical descriptions of a dynamic development of symptoms over time, involving space perception, specific bodily sensations, loss of control, avoidance of certain environments, and the forming of behavioral habits, however, neuroscientific approaches offer snapshot pictures (literally showing photos of typical agoraphobic situations to patients in fMRI) of neural activations, namely hyperactivation of the ventral striatum, insula, amygdala, and hippocampal areas (Wittmann et al., 2011, 2014).

Although contributing to treatment and an explanation of agoraphobia, CBT, along with the correlated neuroscience tend to treat the subject and her surroundings in purely mechanical-causal terms. This is problematic in at least two respects. First, no attention is given to the way in which (inter)subjectivity and spatiality are co-constitutively organized and formed in a meaningful fashion. To the contrary, spatiality is thought of as being a largely neutral canvas, an already formed container, against which the agoraphobic person needs to restructure their way of thinking (Martin and Dahlen, 2005). Second, the lack of attention to the lived experience of spatiality fails to capture the pervasive importance a loss of SA and SO plays in the development of agoraphobic anxiety. Spatiality, for example, is understood as a mere background, which provokes and stimulates an anxiety and sense of panic that ultimately derives from the subject's misinterpretation of the world (Gloster et al., 2013). This overlooks both the rich and relational way in which anxiety is formed, and also fails to consider that the “disorder” involved in agoraphobia involves as much a disorder in spatial experience (or the experience of space as an action space), as a disorder in the SA.

A phenomenological approach to agoraphobic anxiety is helpful here in attending to these oversights (Trigg, 2013a,b, 2016a, in press). As is evident in the preceding analysis of schizophrenia, a phenomenological perspective on anomalous experience reveals not only that SA and SO are integral to a sense of self, but also that a disruption in both SA and AO is not simply an intra-corporeal or intra-psychic occurrence, but instead involves a certain dynamical structure that includes brain, body, and physical and social environments. As such, disruption in SA is not a localized event, but is instead taken up in a disturbance of selfhood more broadly. In the section that follows, we will frame this understanding in terms of the *bodily-inhibition* model of anxiety.

In non-pathological experiences of subjectivity, we experience ourselves for the most part as unified agents. That is to say, we have a prereflective sense of ourselves as both the cause of our bodily movement and also a prereflective sense of being the subject of those movements. Furthermore, in everyday existence SA and SO cohere. As we have seen in the case of schizophrenia, this coincidence of agency and ownership is not absolute. Agoraphobic anxiety affords us another inroad to see SA and SO as integral to understanding both a loss of control and the related disturbance of selfhood. Indeed, in the clinical literature, disturbances of agency, selfhood, and control are presented as being interdependently related. “Agoraphobia,” so Capps and Ochs writes, “is intimately tied to a deep sense of the absence of control over one's feelings and actions” (Capps and Ochs, 1997, p. 152). Likewise, Barlow writes of the “core of anxiety” as involving “the sense of a lack of control” (Barlow, 2002, p. xiii). This loss of control in cases of agoraphobic anxiety is evident in at least two ways: Bodily motricity and bodily objectification.

“Bodily motricity” refers to the body's action-oriented power to project intention into the world in a movement of spontaneity and possibility (Merleau-Ponty, 2012). In this regard it is, from a phenomenological perspective, the general source of SA. Normally we move through the world without significant obstruction. Our bodily experiences and our sense of self cohere, such that we have a prereflective sense that the body as agentive, rather than as “an assemblage of organs juxtaposed in space.” This agentive body is an “indivisible possession,” united and integrated (Merleau-Ponty, 2012,p. 100). In most cases, this capacity of our bodily existence allows us to be situated in the world without the need for reflective or abstract thought. Moreover, in normal instances of bodily action, the spatiality of the world is not divided and dissected into fragmented parts, but instead unfolds in uniform with the synthesis of the body; that is, as a whole. In this respect, background and foreground do not form a binary division, but instead unfold and overlap with one another (p. 113). The result of the body's motricity is that our body operates according to a certain logic, which, whilst not always available to us in reflection, nevertheless serves to underscore a temporal and spatial unity operational “beneath intelligence and perception” (p. 137).

In its everyday motricity the body tends to efface itself, remaining tacitly in the background (Gallagher, 1986; Leder, 1990). This is not an indication of its insignificance, but a marker of its irreducible cohesion and integrity. At the same time the body is an object that we can reflect upon. At times, this reflective stance on the body is employed in a self-conscious manner, such as when I am injured and assess the wound in a critical manner (Legrand, 2007). At other times, my body becomes an object for me against my own volition, such as when I am ill and feel my body as an impediment to my existence. On other occasions, I might experience a broader alienation from my body, such as when I see a photo of myself and fail to identify with the subject captured in the frame. In these moments, we may well have an experience of the body as somehow distinct, other, or *thinglike* (Merleau-Ponty, 1965, p. 209). That the body appears for me as different or even alien does not, of course, attest to substance dualism. Rather, the body's apparent distinction is maintained as a certain affective relation I have to my body. In general, these movements of self-alienation and bodily objectification are brief, and are often consolidated into a unified and relatively coherent sense of self that includes SA and SO, which accompanies us throughout the contingences and ambiguities of our perceptual existence.

Agoraphobia provides us with a different story of the motricity and objectification of the body. In distinction to the normal experience of spatiality and SA, where the body provides a forward-looking *I can*, a trustworthy center of orientation actively engaged with the affordances that surround it, the agoraphobic person's bodily experience of space and agency is marked by hesitancy, disquiet, and a lack of trust in how he or she will respond to an unpredictable or unfamiliar situation (Trigg, 2016a). If the subject is able to move in the world, then it is thanks only to the construction of a rigidly established set of habits and patterns. By way of an illustration, consider several of the motifs appearing in Westphal's case studies: “He cannot visit the zoo in Charlottenburg, because there are no houses” (Knapp, 1988, p. 60); “When in the company of a friend—he then experiences no fear of crossing spaces …The crossing of spaces becomes easier when he stays next to a moving vehicle” (p. 66); “A cane or umbrella in his hand often makes the crossing easier” (p. 70). These examples reveal the highly structured and always conditional way in which people prone to agoraphobia move through the world. Lacking the freedom and agency often taken for granted in bodily existence, the subject has a tendency to rely on a proximity to familiar objects (the home), a means of escape (the car), or a prop employed to forge a spatiality of his or her own (the cane). In each case, the inevitable failure to maintain this tightly woven yet precarious grip on control leads to anxiety. When anxiety emerges, then it does so in the form of what we are calling *bodily inhibition*, and with it a diminished SA.

## The bodily-inhibition model of anxiety

In the notion of *bodily inhibition*, we include two components central to agoraphobic anxiety: A diminishment or disruption in SA and a partial disruption in SO. We maintain that each of these components leads to a broader destabilization in the integrity of selfhood. Moreover, there is evidently a circular relation between (i) anxiety causing a disruption in SA and SO, and (ii) further anxiety being provoked by the loss of SA.

In cases of agoraphobia, anxiety not only causes disruptions in SA and SO, but also exacerbates existing anxiety conditions. This is clear in at least two ways. First, the disruption in SA is related to a shift in bodily motricity. The agoraphobic person's hesitant or inhibited movement in the world often involves an adjoining awareness that anxious sensations and movements originate less from the agent as an integral and unified subject, and more from the body as an autonomous thing (which involves a modulation of SO). As a result, the subject experiences the onset of anxiety (and thus the inhibition of the body) as if coming from nowhere and without any apparent rationale, as Westphal reports on one case study: “He is absolutely unable to offer a specific reason for his feeling of anxiety; it is just there despite all reasoning” (p. 66). Westphal goes on to mention that in all cases “[the patients] absolutely do not know the reasons for this fear. It comes by itself; a sudden occurring, strange thing” (p. 73). The absence of reason explaining the behavior of the agoraphobic person has a critical outcome: She or he experiences the inhibition of movement as being caused by the body as a *thing* rather than as an agentive center of subjectivity. A pattern can be mapped accordingly: (i) sensations of anxiety are experienced as if deriving from nowhere, disrupting agentive self-identification; (ii) the resultant consequence is that patients experience the affected body as not entirely their own; (iii) finally, this partial (but never absolute) loss of SO entails a more generalized disturbance in selfhood.

In such cases, as a thing divorced from self-experience (lacking SO), the body is often presented as having a certain degree of autonomy. Such a body can only be “trusted” in certain situations, and it is for this reason that subjects who often speak of an anxiety over loss of control [as when they fear “becom(ing) crazed and, in panic, jump(ing) over the rail” to a drop below] often invoke the body as having autonomy from the self (Goldstein and Chambless, 1982, p. 131). The American composer and sufferer of agoraphobia Allen Shawn writes as follows of the experience of coming to a standstill when faced with an open space: “If you are very attuned to sensations in your legs, you will notice that they seem to have a mind of their own.…The flight impulse is felt keenly in the legs; it feels almost as if your very limbs were demanding that you run” (Shawn, 2008, p. 119). Here, we have a striking example of the concordance between a lack of bodily motricity (disruption in SA) and bodily objectification (disruption in SO), such that inhibition in movement results in an alienation from the body and the body's potential action. In cases of agoraphobic anxiety, it is not that *I* am the one running from danger, but rather it is *the* legs that are instructing *me* to run. In coming to standstill on a road exposed to wide fields, the legs present themselves as discernible “things” in the world, impeding the experience of the body as “one's own.” In this case the SO for the legs and for the resulting action is disturbed. As bodily motricity ceases to be an *I can* and instead becomes an *I cannot*, so the body becomes partially distinct from the self.

The body that is inhibited by anxiety is a body that renders SO ambiguous. Whilst there is no doubt that it is I who am undergoing and enduring the experience of agoraphobic anxiety, there is nevertheless a parallel uncertainty as to what extent the body and its actions are irreducibly *mine*. In the case of Shawn's illustration, if the body as a whole remains constitutive of his sense of self, then the legs simultaneously contest this sense; neither entirely disowned nor owned; individual body parts instead assume an uncanny quality reflective of a broader disintegration of self during anxiety (Trigg, 2014).

## Anxiety, intersubjectivity, and sense of self

The phenomenological analysis of schizophrenic self-experience—the conception of *ipseity-disturbance* as central to the schizoid pathologies—is instructive in shedding light on disturbances in agoraphobic anxiety In effect, there are close parallels to be drawn between the *ipseity-disturbance* model of schizophrenia and what we are calling the *bodily-inhibition* model of anxiety. Both models involve a heightened self-reflexivity (hyperreflexivity), in which things that are normally taken-for-granted—not least the body's physiological processes—become focal points of attention. In cases of agoraphobic anxiety, this self-reflexivity can be framed as a constant vigilance toward unfamiliar bodily sensations (Trigg, 2016a).

For the sake of the present paper, we are focusing on similarities between schizophrenia and agoraphobic anxiety. Nevertheless, there are also clear qualitative differences between these models. One such difference concerns the temporal structure of these disorders. As understood from the *ipseity-disturbance* model, the difference between cases of schizophrenia and agoraphobic anxiety concerns the temporality involved in the diminishment of self-awareness. If schizophrenia involves the sustained diminishment of a “sense of basic self-presence, the implicit sense of existing as a vital and self-possessed subject of awareness” (Sass and Parnas, 2003, p. 428), in cases of agoraphobic anxiety, this diminishment in self-presence is momentary. The rhythm of agoraphobia is neither homogenous nor uniform, but instead punctuated by moments of bodily integrity, spatial coherence, and self-presence alongside moments of bodily disintegration, spatial incoherence, and self-alienation. Indeed, it is precisely for these reasons that the agoraphobic person divides space into safe/danger and familiar/unfamiliar regions. So long as dangerous and unfamiliar spaces can be avoided, the subject can function in a “normal” fashion.

The idea that the “normal” or healthy self is contemporary with the agoraphobic self is instructive. Of one patient, Westphal notes, “with the exception of (being unable to cross open spaces without anxiety), he likes to believe he is healthy” (Knapp, 1988, p. 63). Likewise, speaking of being in “secure surroundings,” Shawn reflects on how he feels himself to be “normal”: “I even pretend to myself that my ‘personality’ is somehow incompatible with agoraphobia.…Agoraphobia *is* at odds with the tone of some of what I do. I am not wary in every domain” (p. 119–120). Shawn's “normal” personality is the one able to assume the role of a performing pianist, able to face a “hostile audience,” courageous enough to posit a “minority view” at a faculty meeting, and tolerant of “good and bad reviews” of his work (p. 120). Moreover, the “normal” articulation of selfhood is one that is able to circumnavigate the dense but familiar streets of Manhattan without the incursion of anxiety. This “normal” self, then, is precisely defined by a strong sense of being both the originator and controller of bodily movement (SA) coupled with a tacit sense that it is *I* who am undergoing that movement (SO). Agoraphobia is thus patently at odds with this self-presentation of agency and ownership given that the condition is marked by a self-alienation from the body (and thus the world) brought about by a doubt over who/what is inhibiting movement.

With this in mind, we can begin to see how the onset of agoraphobic symptoms marks a broader disturbance in bodily selfhood. If the spatiality of the world is cut up into different regions, then something similar in the index of temporality is true of the body. A person suffering from agoraphobia tends to treat their body as either *owned* when movement is experienced as deriving from the self, or otherwise partially *disowned* when movement feels as though it is inhibited or caused by the autonomy of the body itself.

Notably, the dissection of the body and the world into normal/anxious, owned/disowned, and safe/dangerous categories extends to intersubjectivity, too. Other people are present not as innocuous bystanders or incidental aspects within the world of the agoraphobia, but as constituents in a sense of self and contributors to a loss of self. In the case of the former, the role of the “trusted person” assumes a defining importance in the subject's ability to traverse space without anxiety. In the company of the trusted person, there is an increase in SA. Allen Shawn's ability to cross a wide-open space is assisted not simply by the presence of “safety items” (Xanax, ginger all, and a cell phone) but also by the presence of his companion, who “coaxes” him with the offer of a kiss as a “reward” (Shawn, 2008, p. 118). In the same way that the car, umbrella, and proximity to home serve as “escape routes,” so the same is true of the trusted person who accompanies the agoraphobic person in their anxiety. Their presence signals a familiarity, constancy, and understanding lacking in an otherwise precarious experience of the world (Trigg, 2013a). Barlow describes a “safe person” in the following respect: “A safe person is commonly a significant other whose company enables the patient to feel more comfortable going places than he or she can be either alone or with other people. Usually, this person is considered “safe” because he or she knows about the panic attacks” (Barlow, 2002, p. 343). Having knowledge of the patient's panic attacks not only disarms the efficacy of the panic attack but also provides a legitimate context to manage anxiety should the subject be “incapacitated by panic” (p. 343). As a result, in the company of the trusted other, the agoraphobic person is able to maintain a stronger SA, and an intact SO, than if he or she were alone.

Other people do not always assuage the experience of anxiety; they can also amplify and reinforce an already existing anxiety and rob the subject of the SO (through a process of objectification) and thence of SA. Clinical research on the role of other people in the development of agoraphobic anxiety suggests that the gaze of other people is a significant factor in precipitating the onset of panic (cf. Davidson, 2002). Indeed, a heightened self-consciousness concerning how other people perceive the subject is consistent with the ongoing desire to maintain the self-presentation of being a “normal” and “healthy” individual both to oneself and to others (Vincent, 1919). In this respect, other people are a critical problem for agoraphobic people. Whereas, spatial routes and bodily habits can be controlled to some extent by developing a set of habitual patterns that render perceptual experience predictable, exerting control over how other people perceive us remains impossible. In this respect, the very centrality of the home as the safe place *par excellence* is predicated on its function as concealing the look of the other, as Joyce Davidson notes, “[s]ufferers' homes are frequently organized to minimize the fear of the look” (Davidson, 2003, p. 84). Unlike inanimate props such as cars and umbrellas, other people are not simply objects for our own use, but also perceive us as objects in the world (Sartre, 1998). As objectified by the look of the other, the attempt at maintaining a presentation of being “normal” for the subject proves contentious. Through the look of the other, the attempt at concealing anxiety through adhering to a ritualized and regulated life risks being detected, and in being detected, the very anxiety that the subject seeks to mask from the world in turn becomes an object of interrogation for the other person.

## Conclusion

In this paper we have explicated the distinctions and connections between the prereflective experience and the reflective AA and ownership, within the context of anomalous bodily experiences in schizophrenia and agoraphobia. We've shown that these phenomena are more complex and ambiguous than usually thought, both in terms of their neuronal bases and in terms of their relations with extra-neural factors. We suggested that in cases of schizophrenic delusions of control, disruptions in SA at the level of first-order experience may lead to problems with the reflective attributions of both agency and ownership. Those who suffer from such delusions at times experience their thoughts, actions, and bodily movements as alien and controlled by another agent.

In the case of agoraphobia, disruptions in SA reveal the dynamic and relational structure of this condition. In terms of first-person experience, subjects with phobic anxiety tend to mistrust their own response to the world, feeling their bodies could give way at any point, thus positioning the locus of control outside of selfhood. In such cases, loss of SA leads to a partial loss of SO for body and bodily action. In the context of intersubjective relations, for people suffering from agoraphobia, the encroachment of other people into one's own space leads to anxiety, generating an experience of the body as both my own and not my own concurrently.

The underlying dynamics of these disorders with respect to SA and SO and how they fit into the pattern of self-experience and its disruption are different. It's clear, however, that in both disorders SA and SO cannot be considered in isolation from one another, but instead form an interdependent pairing.

We also hope to have shown the relevance of phenomenological accounts of schizophrenia and agoraphobia, and that purely causal-mechanistic explanations may not be able to capture everything of importance in these disorders. In focusing on disruptions in SA and SO, we have not said enough about the responses to the significantly alien character of the experiences. To such experiences there are at least two possible responses corresponding to the two conditions that we have discussed: (1) anxiety and a retreating reaction against the alien nature of the experience, generating temporally intermittent variations in experience, and in some cases the possibility of a reflective management; or (2) a response that continues and builds contact with the alien experience—a following along in which the subject is drawn into a more permanent delusional withdrawal of meaning even as he continues to try to make sense of it.

## Author contributions

All authors listed, have made substantial, direct and intellectual contribution to the work, and approved it for publication.

## Funding

Research for this paper was supported by the Humboldt Foundation's Anneliese Maier Research Award, and a Visiting Research Fellowship at Kebel College, Oxford (SG) and the Marie Curie International Outgoing Fellowship, FP7-PEOPLE-2013-IOF 624968 (DT).

### Conflict of interest statement

The authors declare that the research was conducted in the absence of any commercial or financial relationships that could be construed as a potential conflict of interest.

## Footnotes

^1^We limit our discussion of anxiety to agoraphobia; this is a result of constraints of space and thematic scope. No doubt, social anxiety, and generalized anxiety each involve a specific and complex conceptualization of SA and ownership. Such anxieties are likely to have overlaps with that of agoraphobia, but nevertheless merit a separate investigation.

^2^We're assuming that neither the action nor SA is a momentary phenomenon but extends over time.

^3^Christensen et al. (2016) have shown that fear and anger can reduce an implicit measure (action binding) for SA.

^4^A study by Caspar et al. (2016), for example, shows that SA, measured by implicit intentional binding, decreases when agents act under orders.

^5^It may be that in delusions of control the schizophrenic starts to experience what Merleau-Ponty has called the impersonal that subtends our personal life: “if I wanted to express perceptual experience with precision, I would have to say that one perceives in me, and not that I perceive. Every sensation includes a seed of dream or depersonalization, as we experience through this sort of stupor into which it puts us when we truly live at the level of sensation” (Merleau-Ponty, 2012, p. 249/223). We typically do not live at the level of sensation, however; we are typically perceivers and agents living in the world. In schizophrenia, this natural attitude can be disrupted. As Merleau-Ponty suggests, one may find a similar movement toward the impersonal or pure sensibility in art. Levinas suggests something similar. Art leaves “the level of perception so as to reinstate sensation” (Levinas, 2001, p. 85/47); it allows us to “wander about in sensation” and to return to the “impersonality of the elements” (Levinas, 2001, p. 85–86/47). Merleau-Ponty attributes these kinds of experiences to Cézanne, who he describes as schizothymic. Cézanne's paintings reveal the “base of inhuman nature” that our human agency hides from us (Merleau-Ponty, 1964, p. 76)—an “unfamiliar” world the experience of which may lead to an uncomfortable anxiety.

^6^The idea that this is a cognitive restructuring which involves a change in beliefs may suggest a response to Bayne and Pacherie's (2007) question about how a belief might be related to changes in ANS. This, as well as the exposure therapy mentioned below, suggests that the C and the B in CBT are not separable, but, at the very least, and consistent with concepts of embodied cognition, integrated.

## References

[B1] Albahari M. (2006). Analytic Buddhism: The Two-tiered Illusion of Self. New York, NY: Palgrave Macmillan.

[B2] Aslam N. (2012). Management of panic anxiety with agoraphobia by using cognitive behavior therapy. Indian J. Psychol. Med. 34, 79–81. 10.4103/0253-7176.96166 22661814 PMC3361850

[B3] Barlow D. (2002). Anxiety and its Disorders. New York, NY: Guilford Press.

[B4] Bayne T. Pacherie E. (2004). Bottom-up or top-down: campbell's rationalist account of monothematic delusions. Philos. Psychiatry Psychol. 11, 1–11. 10.1371/journal.pone.0145275 26735490 PMC4703393

[B5] Bayne T. Pacherie E. (2007). Narrators and comparators: the architecture of agentive self-awareness. Synthese 15, 475–491. 10.1007/s11229-007-9239-9

[B6] Bermúdez J. L. (2011). Bodily awareness and self-consciousness, in The Oxford Handbook of the Self, ed Gallagher S. (Oxford: Oxford University Press), 157–179.

[B7] Billon A. (2013). Does consciousness entail subjectivity? The puzzle of thought insertion. Philos. Psychol. 26, 291–314. 10.1080/09515089.2011.625117

[B8] Billon A. Kriegel U. (2014). Jaspers' dilemma: the psychopathological challenge to subjectivity theories of consciousness, in Disturbed Consciousness, ed Gennaro R. (Cambridge, MA: MIT Press), 29–54.

[B9] Bortolotti L. Broome M. (2009). A role for ownership and authorship in the analysis of thought insertion. Phenomenol. Cogn. Sci. 8, 205–224. 10.1007/s11097-008-9109-z

[B10] Buhrmann T. Di Paolo E. (2015). The sense of agency–a phenomenological consequence of enacting sensorimotor schemes. Phenomenol. Cogn. Sci. 10.1007/s11097-015-9446-7. [Epub ahead of print].

[B11] Cahill C. Frith C. (1996). False perceptions or false beliefs? Hallucinations and delusions in schizophrenia, in Method In Madness: Case Studies In Cognitive Neuropsychiatry, eds Halligan P. W. Marshall J. C. (Hove: Psychology Press), 267–291.

[B12] Campbell J. (2002). The ownership of thoughts. Philos. Psychol. Psychiatry 9, 35–39. 10.1353/ppp.2003.0001

[B13] Capps L. Ochs E. (1997). Constructing Panic: The Discourse of Agoraphobia. Boston, MA: Harvard University Press.

[B14] Caspar E. A. Christensen J. F. Cleeremans A. Haggard P. (2016). Coercion changes the sense of agency in the human brain. Curr. Biol. 26, 585–592. 10.1016/j.cub.2015.12.067 26898470 PMC4791480

[B15] Caspar E. A. De Beir A. Magalhaes De Saldanha Da Gama P. A. Yernaux F. Cleeremans A. Vanderborght B. (2015). New frontiers in the rubber hand experiment: when a robotic hand becomes one's own. Behav. Res. Methods 47, 744–755. 10.3758/s13428-014-0498-3 24942249

[B16] Christensen J. F. Yoshie M. Di Costa S. Haggard P. (2016). Emotional valence, sense of agency and responsibility: a study using intentional binding. Conscious. Cogn. 43, 1–10. 10.1016/j.concog.2016.02.016 27174794

[B17] Clark D. M. (1988). A cognitive model of panic attacks, in Panic: Psychological Perspectives, eds Rachman S. Maser J. D. (Hillsdale, NJ: Erblaum), 71–90.

[B18] Daprati E. Franck N. Georgieff N. Proust J. Pacherie E. Dalery J.. (1997). Looking for the agent: an investigation into consciousness of action and self-consciousness in schizophrenic patients. Cognition 65, 71–86. 10.1016/S0010-0277(97)00039-5 9455171

[B19] David N. Newen A. Vogeley K. (2008). The “sense of agency” and its underlying cognitive and neural mechanisms. Conscious. Cogn. 17, 523–534. 10.1016/j.concog.2008.03.004 18424080

[B20] Davidson J. (2002). ‘Putting on a face’: sartre, goffman, and agoraphobic anxiety in social space. Environ. Planning D 21, 107–122. 10.1068/d45j

[B21] Davidson J. (2003). Phobic Geographies: The Phenomenology of Spatial Identity. London: Ashgate Press.

[B22] Davies M. Coltheart M. Langdon R. Breen N. (2001). Monothematic delusions: towards a two-factor account. Philos. Psychiatry Psychol. 8, 133–158. 10.1353/ppp.2001.0007

[B23] de Vignemont F. (2007). Habeas corpus: the sense of ownership of one's own body. Mind Lang. 22, 427–449. 10.1111/j.1468-0017.2007.00315.x

[B24] de Vignemont F. (2013). The mark of bodily ownership. Analysis 73, 643–651.

[B25] de Vignemont F. Fourneret P. (2004). The sense of agency: a philosophical and empirical review of the “Who” system. Conscious. Cogn. 3, 1–19. 10.1057/9780230800540 14990237

[B26] Edelman R. E. Chambless D. L. (1993). Compliance during sessions and homework in exposure-based treatment of agoraphobia. Behav. Res. Ther. 31, 767–773. 10.1016/0005-7967(93)90007-H 8257408

[B27] Ellis H. D. Young A. W. (1990). Accounting for delusional misidentifications. Br. J. Psychiatry 157, 239–248. 10.1192/bjp.157.2.239 2224375

[B28] Farrer C. Frith C. D. (2002). Experiencing oneself vs. another person as being the cause of an action: the neural correlates of the experience of agency. NeuroImage 15, 596–603. 10.1006/nimg.2001.1009 11848702

[B29] Friston K. (2011). What is optimal about motor control? Neuron 72, 488–498. 10.1016/j.neuron.2011.10.018 22078508

[B30] Frith C. D. (1992). The Cognitive Neuropsychology of Schizophrenia. Hillsdale, NJ: Lawrence Erlbaum Associates.

[B31] Frith C. D. Blakemore S. Wolpert D. (2000). Abnormalities in the awareness and control of action. Philos. Trans. Roy. Soc. Lond. 355, 1771–1788. 10.1098/rstb.2000.0734 11205340 PMC1692910

[B32] Gallagher S. (1986). Lived body and environment. Res. Phenomenol. 16, 139–170. 10.1163/156916486X00103

[B33] Gallagher S. (2000a). Philosophical conceptions of the self: implications for cognitive science. Trends Cogn. Sci. 4, 14–21. 10.1016/S1364-6613(99)01417-5 10637618

[B34] Gallagher S. (2000b). Self-reference and schizophrenia: a cognitive model of immunity to error through misidentification, in Exploring the Self: Philosophical and Psychopathological Perspectives on Self-experience, ed Zahavi D. (Amsterdam; Philadelphia, PA: John Benjamins), 203–239.

[B35] Gallagher S. (2004). Neurocognitive models of schizophrenia: a neurophenomenological critique. Psychopathology 37, 8–19. 10.1159/000077014 14988645

[B36] Gallagher S. (2005). How the Body Shapes the Mind. Oxford: Oxford University Press.

[B37] Gallagher S. (2007). Sense of agency and higher-order cognition: levels of explanation for schizophrenia. Cogn. Semiotics 0 32–48.

[B38] Gallagher S. (2011). Time in action, in Oxford Handbook on Time, ed Callender C. (Oxford: Oxford University Press), 419–437.

[B39] Gallagher S. (2012). Multiple aspects in the sense of agency. N. Ideas Psychol. 30, 15–31. 10.1016/j.newideapsych.2010.03.003

[B40] Gallagher S. (2014). The cruel and unusual phenomenology of solitary confinement. Front. Psychol. 5:585. 10.3389/fpsyg.2014.00585 24971072 PMC4054665

[B41] Gallagher S. (2015). Relations between agency and ownership in the case of schizophrenic thought insertion. Rev. Philos. Psychol. 6, 865–879. 10.1007/s13164-014-0222-3

[B42] Garety P. A. Kuipers E. Fowler D. Freeman D. Bebbington P. E. (2001). A cognitive model of the positive symptoms of psychosis. Psychol. Med. 31, 189–195. 10.1017/S0033291701003312 11232907

[B43] Georgieff N. Jeannerod M. (1998). Beyond consciousness of external events: a ‘who’ system for consciousness of action and self-consciousness. Conscious. Cogn. 7, 465–477. 10.1006/ccog.1998.0367 9787056

[B44] Gloster A. T. Hauke C. Höfler M. Einsle F. Fydrich T. Hamm A.. (2013). Long-term stability of cognitive behavioral therapy effects for panic disorder with agoraphobia: a two-year follow-up study. Behav. Res. Ther. 51, 830–839. 10.1016/j.brat.2013.09.009 24184430

[B45] Goldstein A. Chambless D. (1982). Agoraphobia: Multiple Perspectives on Theory and Treatment. (Chichester: Wiley and Sons).

[B46] Graham G. Stephens G. L. (eds.). (1994). Mind and mine, in Philosophical Psychopathology, (Cambridge, MA: MIT Press), 91–109.

[B47] Grünbaum T. (2015). The feeling of agency hypothesis: a critique. Synthese 92, 3313–3337. 10.1007/s11229-015-0704-6

[B48] Haggard P. (2005). Conscious intention and motor cognition. Trends Cogn. Sci. 9, 290–95. 10.1016/j.tics.2005.04.012 15925808

[B49] Haggard P. Tsakiris M. (2009). The experience of agency feelings, judgments, and responsibility. Curr. Dir. Psychol. Sci. 18, 242–246. 10.1111/j.1467-8721.2009.01644.x

[B50] Hahn T. Kircher T. Straube B. Wittchen H. U. Konrad C. Ströhle A.. (2015). Predicting treatment response to cognitive behavioral therapy in panic disorder with agoraphobia by integrating local neural information. JAMA Psychiatry 72, 68–74. 10.1001/jamapsychiatry.2014.1741 25409415

[B51] Hohwy J. Rosenberg R. (2005). Unusual experiences, reality testing and delusions of alien control. Mind Lang. 20, 141–162. 10.1111/j.0268-1064.2005.00280.x

[B52] Jeannerod M. (2009). The sense of agency and its disturbances in schizophrenia: a reappraisal. Exp. Brain Res. 192, 527–532. 10.1007/s00221-008-1533-3 18709365

[B53] Knapp T. (1988). Westphal's “Die Agoraphobie” with Commentary: The Beginnings of Agoraphobia. Lanham: University Press of America.

[B54] Langland-Hassan P. (2008). Fractured phenomenologies: thought insertion, inner speech, and the puzzle of extraneity. Mind Lang. 23, 369–401. 10.1111/j.1468-0017.2008.00348.x

[B55] Leder D. (1990). The Absent Body. Chicago, IL; London: University of Chicago Press.

[B56] Legrand D. (2007). Pre-reflective self-consciousness: on being bodily in the world. Janus Head. 9, 493–519. 10.1016/j.concog.2007.04.002

[B57] Levinas E. (2001). Existence and Existents, Trans. A. Lingis. Pittsburgh: Duquesne University Press.

[B58] Lueken U. Straube B. Konrad C. Wittchen H. U. Ströhle A. Wittmann A.. (2013). Neural substrates of treatment response to cognitive-behavioral therapy in panic disorder with agoraphobia. Am. J. Psychiatry 170, 1345–1355. 10.1176/appi.ajp.2013.12111484 23982225

[B59] Marks I. (1987). Fears, Phobias, and Rituals: Panic, Anxiety, and their Disorders. Oxford: Oxford University Press.

[B60] Martin R. Dahlen E. (2005). Cognitive emotion regulation in the prediction of depression, anxiety, stress, and anger. Pers. Indiv. Dif. 39, 1249–1260. 10.1016/j.paid.2005.06.004

[B61] Merleau-Ponty M. (1964). Sense and Non-sense. Evanston, IL: Northwestern University Press.

[B62] Merleau-Ponty M. (1965). The Structure of Behavior. Trans. Alden Fisher. Boston, MA: Beacon Press.

[B63] Merleau-Ponty M. (2012). Phenomenology of Perception. Trans. Donald Landes. New York, NY; London: Routledge.

[B64] Meyer V. Gelder M. G. (1963). Behaviour therapy and phobic disorders. Br. J. Psychiatry 109, 19–28. 10.1192/bjp.109.458.19 13935321

[B65] Mundale J. Gallagher S. (2009). Delusional experience, in Oxford Handbook of Philosophy and Neuroscience, ed Bickle J. (Oxford: Oxford University Press), 513–521.

[B66] Pacherie E. (2006). Towards a dynamic theory of intentions, in Does Consciousness Cause Behavior? An Investigation of the Nature of Volition, eds Pockett S. Banks W. P. Gallagher S. (Cambridge, MA: MIT Press), 145–167.

[B67] Pacherie E. (2008). The phenomenology of action: a conceptual framework. Cognition 107, 179–217. 10.1016/j.cognition.2007.09.003 17950720

[B68] Pacherie E. Green M. Bayne T. (2006). Phenomenology and delusions: who put the ‘alien’ in alien control? Conscious. Cogn. 15, 566–577. 10.1016/j.concog.2005.11.008 16403655

[B69] Park S. Nasrallah H. (2014). The varieties of anomalous self experiences in schizophrenia: splitting of the mind at a crossroad. Schizophr. Res. 152, 1–4. 10.1016/j.schres.2013.11.036 24332407

[B70] Parnas J. Sass L. (2011). The structure of self-consciousness in schizophrenia, in The Oxford Handbook of the Self, ed Gallagher S. (Oxford: Oxford University Press), 521–546.

[B71] Sartre J. P. (1998). Being and Nothingness. Trans. Hazel Barnes. London; New York, NY: Routledge.

[B72] Sass L. (2014). Self-disturbance and schizophrenia: structure, specificity, pathogenesis. Schizophr. Res. 152, 5–11. 10.1016/j.schres.2013.05.017 23773296

[B73] Sass L. Parnas J. (2003). Schizophrenia, consciousness, and the self. Schizophr. Bull. 29, 427–444. 10.1093/oxfordjournals.schbul.a007017 14609238

[B74] Shawn A. (2008). Wish I Could Be There: Notes from a Phobic Life. New York, NY: Penguin.

[B75] Sierra M. (2009). Depersonalization: A New Look at a Neglected Syndrome. Cambridge: Cambridge University Press.

[B76] Singh J. R. Knight T. Rosenlicht N. Kotun J. M. Beckley D. J. Woods D. L. (1992). Abnormal premovement brain potentials in schizophrenia. Schizophr. Res. 8, 31–41. 10.1016/0920-9964(92)90058-D 1358184

[B77] Stephens G. L. Graham G. (2000). When Self-Consciousness Breaks: Alien Voices and Inserted Thoughts. Cambridge MA: MIT Press.

[B78] Synofzik M. Vosgerau G. Newen A. (2008). Beyond the comparator model: a multifactorial two-step account of agency. Conscious. Cogn. 17, 219–239. 10.1016/j.concog.2007.03.010 17482480

[B79] Trigg D. (2013a). The body of the other: intercorporeality and the phenomenology of agoraphobia. Cont. Philos. Rev. 46, 413–429. 10.1007/s11007-013-9270-0

[B80] Trigg D. (2013b). Bodily moods and unhomely environments: the hermeneutics of agoraphobia, in Interpreting Nature: The Emerging Field of Environmental Hermeneutics. eds Clingerman F. Treanor B. Drenthen M. Ulster D. (New York, NY: Fordham University Press), 160–177.

[B81] Trigg D. (2014). The Thing: a Phenomenology of Horror. Washington, DC: Zero Books.

[B82] Trigg D. (2016a). Topophobia: a Phenomenology of Anxiety. London: Bloomsbury.

[B83] Trigg D. (in press). Agoraphobia, Sartre, the Spatiality of the Other's Look, in Body/Self/Other: Phenomenology of Social Encounters. eds Dolezal L. Petherbridge D. (New York, NY: SUNY).

[B84] Tsakiris M. Bosbach S. Gallagher S. (2007). On agency and body-ownership: phenomenological and neuroscientific reflections. Conscious. Cogn. 16, 645–660. 10.1016/j.concog.2007.05.012 17616469

[B85] Tsakiris M. Haggard P. (2005). Experimenting with the acting self. Cogn. Neuropsychol. 22, 387–407. 10.1080/02643290442000158 21038257

[B86] Tsakiris M. Longo M. R. Haggard P. (2010). Having a body versus moving your body: neural signatures of agency and body-ownership. Neuropsychologia 48, 2740–2749. 10.1016/j.neuropsychologia.2010.05.021 20510255

[B87] Vincent (1919). Confessions of an agoraphobic victim. Am. J. Psychol. 30, 295–299.

[B88] Vinding M. C. Pedersen M. N. Overgaard M. (2013). Unravelling intention: distal intentions increase the subjective sense of agency. Conscious. Cogn. 22, 810–815. 10.1016/j.concog.2013.05.003 23732190

[B89] Vogeley K. Kurthen M. Falkai P. Maier W. (1999). Essential functions of the human self model are implemented in the prefrontal cortex. Conscious. Cogn. 8(3), 343–363. 10.1006/ccog.1999.0394 10487788

[B90] Wittmann A. Schlagenhauf F. Guhn A. Lueken U. Gaehlsdorf C. Stoy M.. (2014). Anticipating agoraphobic situations: the neural correlates of panic disorder with agoraphobia. Psychol. Med. 44, 2385–2396. 10.1017/S0033291713003085 24398049

[B91] Wittmann A. Schlagenhauf F. John T. Guhn A. Rehbein H. Siegmund A.. (2011). A new paradigm (Westphal-Paradigm) to study the neural correlates of panic disorder with agoraphobia. Eur. Arch. Psychiatry Clin. Neurosci. 261, 185–194. 10.1007/s00406-010-0167-1 21113608

[B92] Wolpert D. M. Flanagan J. R. (2001). Motor prediction. Curr. Biol. 11, R729–R732. 10.1371/journal.pone.0156882 11566114

